# Concurrent *Toxoplasma gondii* infection and neuroinflammation in traumatic brain injury patients in a referral hospital in Douala Cameroon

**DOI:** 10.1038/s41598-026-40284-1

**Published:** 2026-03-12

**Authors:** Franklin Chu Buh, Germain Sotoing Taiwe, Andrew I. R. Maas, Mathieu Motah, Ignatius Esene, Firas H. Kobeissy, Vanessa Tita Jugha, Eric Youm, Basil Kum Meh, Kevin W. Wang, Peter J. A. Hutchinson, Irene Ule Ngole Sumbele

**Affiliations:** 1 Department of Animal Biology and Conservation, Faculty of Science, University of Buea, P.O. Box 63, Buea, Cameroon; 2 Department of Neurosurgery, Antwerp University Hospital, University of Antwerp, Edegem, 2000 Belgium; 3 Department of Surgery, Faculty of Medicine and Pharmaceutical Sciences, University of Douala, P.O. Box 2701, Douala, Cameroon; 4 Department of Neurosurgery, Faculty of Health Sciences, University of Bamenda, Bamenda, Cameroon; 5 Department of Biochemistry and Molecular Genetics, Faculty of Medicine, American University of Beirut, Riad El-Solh, P.O. Box 11-0236, Beirut, Lebanon; 6 Holo Healthcare, Nairobi, 00400 Kenya; 7 Center for Neurotrauma, Multiomics & Biomarkers (CNMB), Department of Neurobiology, Neuroscience Institute, Morehouse School of Medicine, 720 Westview Dr SW, Atlanta, GA 30310-1458 USA; 8 Department of Clinical Neuroscience, Cambridge, CB2 0QQ UK

**Keywords:** Inflammatory markers, Neuroinflammation, Neuropathology, Traumatic brain injury, *Toxoplasma gondii*, Neuroscience, Biomarkers, Medical research, Pathogenesis

## Abstract

Studies on murine models show that *Toxoplasma gondii* infection reduces cerebral microvascular perfusion and induces neuroinflammation by activating cerebral endothelial cells, which could affect traumatic brain injury (TBI) outcomes. Whether TBI inflammatory profile and outcomes differ in persons with concurrent latent infection with *Toxoplasma gondii* has not yet been elucidated in TBI patients, constituting the core of this study. Understanding the impact of chronic *T. gondii* infection on neuroinflammation after TBI may ensure better management and prognosis of TBI. This study aimed to investigate the inflammatory profile in TBI patients and investigate the influence of concurrent *T. gondii* infection on inflammatory markers and TBI outcomes. The level of inflammatory markers [interleukin (IL)-1β, IL-6, IL-10, interferon-gamma (INF-ɣ), Tumour necrosis factor-alpha (TNF-α)], and *Toxoplasma gondii* infection were detected in serum obtained < 24 h post-injury. Glasgow Outcome Scale-Extended (GOSE) was used to evaluate the 6-month outcome post-discharge. The Wilcoxon and Kruskal-Wallis’s rank sum tests were used to compare concentrations of inflammatory markers to *T. gondii infection* and TBI outcome. *T. gondii* infection was detected in 33% (52/160) of TBI cases. There was a significant increase (*p* < 0.001) in the concentrations of all inflammatory markers (IM) in TBI patients compared with the healthy controls. Higher levels of IL-6, INF-ɣ, and TNF-α were associated with mortality. Findings from this study show an increase in IM levels for all the *T. gondii-positive* TBI cases, which were significant for IL-1β (*P* < 0.001) and TNF-α (*P* < 0.001). IL-1β and TNF-α, had significantly greater density values, above 30pg/mL and 90pg/mL, respectively, in TBI patients infected with *T. gondii*, compared to greater density values, below 30 pg/mL and 90 pg/mL, respectively, for TBI patients seronegative to *T. gondii.* Concurrent *T. gondii* infection in TBI significantly influenced the inflammation profile of patients. Further multicenter studies with larger sample sizes will provide more insights into *T. gondii* induced neuropathology and prognostication in TBI care.

**Supplementary Information:**

The online version contains supplementary material available at 10.1038/s41598-026-40284-1.

## Introduction

Traumatic brain injury (TBI) is a leading cause of death and disability^1^affecting about 70 million individuals worldwide^2^. It disproportionately affects Low-middle-income countries (LMICs) and the incidence is expected to rise to 14 million by the year 2050^3^. TBIs constitute a huge medical, societal and socio-economic problem worldwide, making understanding of its pathological mechanisms, management and outcome extremely important^4,5^. It is extremely common, with a lifetime prevalence of up to 40% among adults^6^. A TBI prevalence of 34% has been reported in Cameroon^5^. The burden of TBI is on the rise worldwide with Sub-Saharan Africa (SSA) being the focus considering its fast-rising incidence^7,8^. Although its burden is most felt in low-income settings, TBI remains a problem of global health importance. Therefore, understanding the pathobiology aspects of TBI like neuroinflammation, is capital for adequate diagnosis, prognosis, and management^9^.

Neuroinflammation in TBI has been reported as one of the major pathophysiologic mechanisms associated with adverse outcomes as it contributes to brain damage^10–12^. Although microglia play an important role in neuroinflammation as it constitutes the first line of defence in brain injury, it can produce excessive proinflammatory mediators that exacerbate brain damage, and hinder brain repair as well as neurological functional recovery in the secondary cascade^13–16^. Whilst more has been done on the role of neuroinflammation in the aetiology of neurodegenerative disorders^11^, enough emphasis has not been shown on its role in TBI pathology and outcomes in humans. There is a paucity of information in SSA on the serum/plasma concentrations of inflammatory markers (IL-1β, IL-6, IL-10, TNF-α, INF-γ as well as *Toxoplasma gondii* induced neuropathology in TBI. These inflammatory markers including IL-8 are considered biomarkers for TBI diagnosis and prognosis^14^.

Furthermore, neuroinflammation after TBI may be enhanced by infections and *T. gondii* is one of the prominent parasites that infect the Central Nervous System (CNS) with high tropism for the brain. It has neuroinflammatory properties, infects about 1/3 of the world’s population and is capable of existence as a long-life infection^12^.

Toxoplasma parasite was considered in this study because it is very common around the world with a marked increase in infections particularly in low-income settings^15^. Also, it results in chronic low-grade inflammation and altered signalling pathways within the brain, and preliminary clinical evidence suggests that it may be a risk factor for epilepsy^17^. It has been elucidated in mice, that *T. gondii* infection reduces cerebral microvascular perfusion and induces neuroinflammation through activation of cerebral endothelial cells, which could affect TBI outcomes^17,12^. Baker et al.^18^ further demonstrated that *T. gondii-*infected TBI mice had exacerbated pathological responses, reflected in white matter tract abnormalities. Also, the release of glutamate after TBI, coupled with the increased extracellular glutamate in chronic *T. gondii* infection, could result in further excitotoxicity causing progression in secondary insults^19–22^. Although, other parasitic infections like malaria, trypanosomiasis, cysticercosis, schistosomiasis etc. affect the CNS^23^, *T. gondii* has a high global prevalence and has the ability to establish a chronic, latent infection within the brain^18^. Furthermore, several studies have reported post-injury systemic immunosuppression, following neurotrauma (Sribnick et al.^24^; Bouras et al.^25^, 2022; Griffin^26^, 2011; Sharma et al.^27^). Moreover, this post-injury immunosuppression in TBI patients, may reactivate chronic toxoplasmosis, leading to a potentially life-threatening condition. This reactivation is due to various factors impairing the protective cellular immune response such as infections, immunosuppressive therapies, and TBI^28,18^. More attention has been given to other causes of immunosuppression and TBI is often overlooked.

Recent studies have shown that the immune system plays a pivotal role in the pathogenesis of toxoplasmosis by triggering immune cytokines like IL-12, TNF-α, and IFN-γ^29^ and activating a robust immune response which is critical to both host and parasite survival. Thus, the infected host cells produce protective cytokines: IFN**-**γ and TNF, which inhibit *T. gondii* replication and promote its transformation from an active form to tissue cysts^30^.Also, perfusion of Evans blue dye shows increased BBB permeability during chronic *T. gondii* infection, accompanied by reduced blood flow and capillary rarefication which may permit immune cell entry into the brain^31–33^.

Several reports have demonstrated that TBI is associated with neuroinflammation which adversely affects outcomes (cognitive, motor, and emotional abnormalities)^14,12,34,18^. If there is concurrent *T. gondii* infection in these TBI patients, neuroinflammation may be potentiated, resulting in more brain damage and long-term sequelae.

Hence, a thorough understanding of the influence of *T. gondii-induced* neuroinflammation in TBI, especially in LMICs is essential to mitigate TBI-related mortality^34^. Most studies on *T. gondii-*induced neuroinflammation in TBI have used laboratory mice. This novel study among humans seeks to determine the inflammatory profile of patients with mild to severe TBI and the effects of concurrent *T. gondii* infections on the inflammatory markers and TBI outcome.

## Methods

### Study area, design, and period

This prospective cohort study was conducted at the Laquintinie Hospital of Douala (LHD) on acute TBI patients. Douala is the economic capital of Cameroon and is a highly cosmopolitan city with an estimated population of about 3.8 million inhabitants^35,36^. Laquinitinie Hospital Douala (LHD) is a second-category referral hospital located in the heart of Douala. Its mission is to ensure medical care and quantitative and qualitative medico-sanitary responses in major events such as sporting activities, traffic accidents, disasters, or epidemics. It offers many services organized within departments amongst which neurosurgery. The neurosurgical department is well established, and the hospital has three neurosurgeons, a CT Scanner, and a 0.5 Tesla MRI. The hospital was chosen for the study because it received the highest number of trauma cases in the Littoral Region of Cameroon, and probably Cameroon at large. The study was conducted over a period of 13 months, from January 2021 to January 2022.

### Study population and participants

The population of the study consisted of all patients who sustained a mild to severe, TBI, received at the emergency service of the LHD within 24 h of injury.

#### Inclusion criteria

All individuals who were brought to the emergency service of the Laquintinie Hospital Douala who sustained head trauma and for whom written informed consent was obtained from themselves or their family members, were enrolled in the study. All the severities of TBI; mild (GCS 13–15), moderate (GCS 9–12), and severe (GCS 3–8) were included in the study. In addition, fifteen healthy individuals of both sexes were also included as healthy controls. The demographic details and outcomes of this cohort have been reported previously^5,37^.

#### Exclusion criteria

Excluded from the study were individuals with pre-existing neuro-psychiatric problems, and those patients from whom blood samples could not be obtained for various reasons.

### Sampling method

Patients or their families, depending on the severity of TBI and their level of consciousness, were approached to obtain consent to participate in the study. They were briefed on the objective of the study and what was needed from them. Those who gave their consent were enrolled in the study. Information on the sociodemographic, clinical and injury details, were included. Blood samples were collected within 24 h after injury for the determination of the serum concentrations of inflammatory markers IL-1β, IL-6, IL-10, interferon-gamma (INF-ɣ), TNF-α), and *Toxoplasma gondii* infection. Six-month GOSE (favourable vs. unfavourable and survival vs. death) was explored. A favourable outcome was interpreted as a GOSE score from 5 to 8, while an unfavourable outcome was a GOSE score of 1–4^37^. The GOS-E was used to determine the level of recovery 6 months after hospital discharge. The evaluation was done through face-to-face visits or telephone calls.

### Laboratory methods

Blood samples from the study participants were collected into dry tubes and kept for 30 min to an hour for coagulation to take place. The samples were then centrifuged at 3500 rpm for 15 min to obtain the blood serum. Each serum was aliquoted using micro-pipettes and emptied into labelled and coded cryo-tubes. Care was taken to ensure the cryotubes had the same code as the corresponding dry tubes. Serum samples were preserved at -80 °C at the Central Laboratory of the Laquintinie Hospital of Douala until transferred to the Biology Laboratory of the University of Buea, where they were analysed. The methods of sample collection and storage was drawn from the study of Czeiter et al.^38^.

### Measurement of *Toxoplasma gondii* IgG antibodies

The seropositivity to *T. gondii* was measured using Commercial Assay ELISA Kits following the manufacturer’s instructions (MyBioSource INC, USA, MBS494548). This assay is based on the principle; that IgG-specific antibody, if present, binds to the antigen. All unbound materials are washed away, and the enzyme conjugate is added to bind to the antibody-antigen complex, if present. Excess enzyme conjugate is washed off and substrate is added. The plate is incubated to allow the hydrolysis of the substrate by the enzyme. The intensity of the colour generated is proportional to the concentration of IgG-specific antibodies in the sample.

One hundred microliters (100 µL) of diluted sera, calibrator, and controls were dispensed into the appropriate wells. For the reagent blank, a 100 µL sample diluent was emptied into the wells. The holder was tapped to remove air bubbles from the liquid and mixed well, and the plate was incubated for 20 minutes at room temperature. After this, the liquid from all of the wells was removed and the wells were washed three times with 300 µL of 1X wash buffer. The plate was then blotted on absorbance paper. One hundred microliters of enzyme conjugate were added to each well, which was thereafter incubated for 20 minutes at room temperature. The enzyme conjugate was removed from all of the wells, and they were washed three times with 300 µL of 1X wash buffer; then, 100 µL of 3,3’,5,5’-Tetramethylbenzidine (TMB) substrate was added, and the plates were incubated for 10 min at room temperature, after which 100 µL of stop solution was dispensed into each well. The optical density (OD) was read at 450 nm using an ELISA reader within 15 min. The Antibody Index Interpretation was as follows: less than (˂) 0.9: no detectable antibody to *Toxoplasma* IgG by ELISA; 0.9–1.1: borderline positive; >1.1: detectable antibody to *Toxoplasma* IgG by ELISA. The above methods were similar to those by Ebrahimzadeh et al.^39^.

### Measurement of inflammatory markers

The levels of tumour necrosis factor-alpha, interferon-gamma, interleukin 1-beta, interleukin 6 and interleukin 10 in the serum were determined using enzyme-linked immunosorbent assay (ELISA) technique according to the kit manufacturer’s instructions (R&D Systems Biotech, Inc. Minneapolis, USA). This experiment employs the quantitative sandwich enzyme immunoassay technique. Different plates were used for each inflammatory cytokine, respectively. Polyclonal antibodies, specific for human TNF-α, IFN-γ, IL-1β, IL-6, or IL-10, respectively were pre-coated onto 96 wells of microplates. Standards and samples were pipetted into the wells and any TNF-α, IFN-γ, IL-1β, IL-6, or IL-10, present is bound by the immobilized antibody, respectively. After washing away any unbound substances, an enzyme-linked polyclonal antibody specific for each human TNF-α, IFN-γ, IL-1β, IL-6, or IL-10 were added to the respective wells. Following a wash to remove any unbound antibody-enzyme reagent, a substrate solution was added to the wells and colour developed in proportion to the amount of TNF-α, IFN-γ, IL-1β, IL-6, or IL-10 bound in the initial step, respectively. The colour development was stopped, and the optical density of each well was determined, using a Thermo ScientificTM Multiskan FC microplate ELISA photometer setup at 450 nm. Standard curves were used for all the cytokines to ensure the laboratory procedures were well implemented. The procedure for determining inflammatory markers were the same as those used by Baird et al.^40^. The normal serum values were as follows: IL-1β: <12pg/mL, IL-6: <12pg/mL, IL-10: <54pg/mL, TNF-α: <10pg/mL, INF-ɣ: <50pg/mL. These values are like those reported by Wu et al.^41^

### Data analysis

Data collected were cross-checked for any errors. All the questionnaires were given unique codes, and the information was entered into the CSPro 7.6 data mask designed by a statistician. Continuous variables were reported as medians with 25th and 75th percentiles and as means and standard deviations. Categorical variables were described as frequencies and percentages. The Wilcoxon rank sum test and Kruskal−Wallis rank sum tests were used for comparisons between the biomarker concentrations and TBI severity and outcomes. P-values ˂ 0.05 were considered statistically significant. The Benjamini-Hochberg technique was used to adjust p-values in multiple comparisons. Multivariate logistic regression was done to verify if pre-existing *T. gondii* infection could predict mortality post-TBI.

All methods were performed in accordance with the relevant guidelines (see also ethics and consent).

## Results

One hundred and sixty TBI patients were enrolled. Eight, however, were lost to follow-up due to contact difficulties after hospital discharge. Hence, 152 were retained in the outcome analysis.

### Socio-demographic characteristics of participants and TBI severity

Regarding the sociodemographic features of the cohort prospective study, most TBI patients were aged 15–45 years (78%, 125). The least represented age groups were children (˂15 years, 3.8%) and the elderly, (>65, 4.4%). The majority were male subjects (90%, 144). Most of the participants did not finish secondary education (51%, 82) with only 1 (0.6%) who attended post-graduate studies. The most represented professional group was commercial bike riders (27%, 43). A greater number of the TBI cases were single (54%, 84). Regarding TBI severity, mild TBI (41%, 66) was more represented, followed by moderate (34%, 55) and severe TBI (24%, 29). Regarding the healthy controls, majority were in the age group 15–45 years (93%) and males were more represented (80%), as shown in Table 1.

**Table 1:** Sociodemographic characteristics and TBI severity in the prospective cohort.

Characteristic patients	TBI Cases (%)	Healthy Controls
**N**	**160 (100)**	**15 (100)**
**Age in years**	**34 (13)**	
(˂15)	6 (3.8)	
(15–45)	125 (78)	14 (93)
(46–65)	22 (14)	1 (7)
(>65)	7 (4.4)	
**Gender**		
Female	16 (10)	3 (20)
Male	144 (90)	12 (80)
**Education**		NA
Graduate (bachelor’s degree)	19 (12)	
No formal education	9 (5.6)	
Undergraduate	7 (4.4)	
Not known	2 (1.2)	
Post-graduate education	1 (0.6)	
Primary	40 (25)	
Secondary	82 (51)	
**Profession**		NA
Employee in service	30 (19)	
Manual workers	24 (15)	
Bike riders	43 (27)	
Student	16 (10)	
Unemployed	21 (13)	
Others	26 (16)	
**TBI severity**	*N* = 160	**NA**
Mild	66 (41)	
Moderate	55 (34)	
Severe	39 (24)	

TBI: Traumatic Brain Injury.

### *Toxoplasma gondii* infection status and TBI

*T. gondii* infection was recorded in 33% (52/160) of TBI cases. The median age for *T. gondii-positive* patients was 30 (IQR23, IQR39) years. The infection was more common in TBI patients aged 15–45 (77%), and only 3.8% in those aged above 65 years (Table 2).

**Table 2:** *T. gondii* infection status among TBI patients with respect to age, sex and profession.

Characteristic	Overall Median (25th percentile, 75th percentile)	Negative Median (25th percentile, 75th percentile)	Positive Median (25th percentile, 75th percentile)	*P*-value
**N**	**160**	**108**	**52**	
**Age**	32 (26, 39)	32 (27, 38)	30 (23, 39)	0.6
< 15	6 (3.8%)	3 (2.8%)	3 (5.8%)	0.8
15–45	125 (78%)	85 (79%)	40 (77%)	
46–65	22 (14%)	15 (14%)	7 (13%)	
> 65	7 (4.4%)	5 (4.6%)	2 (3.8%)	
**Gender**	**N (%)**	**N (%)**	**N (%)**	0.072
Female	16 (10%)	14 (13%)	2 (4%)	
Male	144 (90%)	94 (87%)	50 (96%)	
**Profession**				0.5
Bike riders	43 (27%)	28 (26%)	15 (28.8%)	
Employee in service	30 (19%)	21 (19%)	9 (17.3%)	
Manual workers	24 (15%)	16 (15%)	8 (15.4%)	
Others	26 (16%)	15 (14%)	11 (21.2%)	
Student	16 (10%)	10 (9%)	6 (11.5%)	
Unemployed	21 (13%)	18 (17%)	3 (5.8%)	

### Inflammatory marker concentrations and TBI cases

The increase in the concentrations of all inflammatory markers evaluated in TBI patients [IL-10: 254 (233, 270), IL-1β: 29 (24, 34), IL-β: 149 (131, 193), INF-ɣ : 85 (81, 91), TNF-α: 77 (69, 92) compared to the healthy controls [37 (32, 42), 7 (7, 8), 10 (10, 11), 28 (27, 28), 6 (5, 7)], was statistically significant (*p* < 0.001) as shown in Table 3.

**Table 3:** A comparison of inflammatory markers by cases and healthycontrols.

Characteristic	TBI cases Median (25th percentile, 75th percentile)	Healthy Controls Median (25th percentile, 75th percentile)	*P*-value
**N**	**160**	**15**	
IL-10 (pg/mL)	254 (233, 270)	37 (32, 42)	< 0.001
IL-1β (pg/mL)	29 (24, 34)	7 (7, 8)	< 0.001
IL-6 (pg/mL)	149 (131, 193)	10 (10, 11)	< 0.001
INF-ɣ (pg/mL)	85 (81, 91)	28 (27, 28)	< 0.001
TNF-α (pg/mL)	77 (69, 92)	6 (5, 7)	< 0.001

Significant at *P* < 0.001.

IL: Interleukin, INF-ɣ: Interferon-gamma, TNF-α: Tumour necrosis factor-alpha, pg: picogram, mL: millilitre, pg/mL: picogram/millilitre.

### Comparison of the neuroinflammatory cytokines concentrations and post-injury time

Comparing neuroinflammatory markers with post-injury time of arrival at the hospital revealed no statistically significant difference (*P* > 0.05) between patients who arrived less than 12 h [IL-10: 255 (235, 271), IL-1β: 29 (24, 34), IL-6: 149 (131, 193), INF-ɣ : 85 (81, 91), and TNF-α: 76 (69, 92)] pg/mL compared to those who arrived between 12 and 24 h [IL-10: 252 (232, 268), IL-1β: 28 (26, 36), IL-6: 156 (142, 191), INF-ɣ : 83 (81, 91), and TNF-α: 79 (69, 92)]pg/mL (Supplementary Table 1).

### Comparison of inflammatory cytokines concentrations and TBI severity

The serum concentrations of the neuroinflammatory markers evaluated increased in the TBI cases. However, these levels did not vary significantly with the severity of TBI. Observations revealed IL-10 and IL-6 values were higher in mild (257 pg/mL and 166 pg/mL) than severe in TBI (250 pg/mL and 153 pg/mL), respectively, as shown in Table 4.

**Table 4:** Concentration of inflammatory cytokines according to the severity ofTBI.

Characteristic	Mild Median (25th percentile, 75th percentile)	Moderate Median (25th percentile, 75th percentile)	Severe Median (25th percentile, 75th percentile)	*P*-value
**N**	**66**	**55**	**39**	
IL-10 (pg/mL)	257 (236, 268)	256 (232, 275)	250 (232, 270)	0.4
IL-1β (pg/mL)	29 (24, 35)	28 (23, 32)	28 (24, 33)	0.3
IL-6 (pg/mL)	166 (142, 198)	146 (129, 184)	153 (135, 196)	0.3
INF-ɣ (pg/mL)	85 (81, 91)	85 (81, 92)	83 (81, 89)	0.8
TNF-α (pg/mL)	78 (70, 93)	74 (68, 92)	79 (64, 92)	0.8

IL: Interleukin, INF-ɣ: Interferon-gamma, TNF-α: Tumour necrosis factor-alpha, pg: picogram, mL: millilitre.

### Comparison of inflammatory cytokine concentrations with normal vs. abnormal CT scans and imaging location

Table 5 below shows slightly higher values of TNF-α: 78 (66, 92) in patients with traumatic intracranial abnormality, compared to 76 (71, 92) within Normal CT. However, there was not a statistically significant difference between cytokine concentrations and imaging outcome (*p* > 0.05) as shown in Table 5. As seen in Table 6 below, cytokine concentrations did not vary significantly with imaging location (*p* > 0.05). However, IL-10 (pg/mL) had highest concentrations in patients with Cerebral contusion (CC) [270 (236, 279)] and intracerebral haemorrhage (ICH) [263 (236, 270). IL-1β (pg/mL) had highest concentrations in ICH [32 (22, 47), CC [29 (26, 33)], and cerebral oedema (COE) [29 (26, 33). IL-6 (pg/mL) had highest concentration in extradural haemorrhage (EDH) [165 (142, 188)]. INF-ɣ (pg/mL) had highest concentration in CC [92 (82, 93)], and TNF-α (pg/mL), highest in COE (81 (71, 93).

**Table 5:** Concentration of cytokines with CT-scan results.

Characteristic	Normal CT *N* = 42	TIC abnormality *N* = 70	*p*-value
IL-10 (pg/mL)	254 (232, 269)	250 (235, 270)	0.5
IL-1β (pg/mL)	28 (22, 34)	28 (25, 31)	0.9
IL-6 (pg/mL)	151 (131, 196)	146 (128, 172)	0.14
INF-ɣ (pg/mL)	85 (82, 91)	83 (81, 90)	0.067
TNF-α (pg/mL)	76 (71, 92)	78 (66, 92)	0.7

IL Interleukin, INF-ɣ: Interferon-gamma, TNF-α: Tumour necrosis factor-alpha, pg/mL: picogram/millilitre, TIC: Traumatic Intracranial..

**Table 6:** Concentration of cytokines with imaging location (type of TBI).

Type of TBI	Cytokines	Other TBI	TBI considered	*p*-value
**EDH**		**N = 50**	**EDH, N = 20**	
	IL-10 (pg/mL)	249(232, 269)	259 (236, 277)	0.427
	IL-1β (pg/mL)	28 (23, 33)	27 (25, 31)	0.882
	IL-6 (pg/mL)	146 (130, 179)	165 (142, 188)	0.427
	INF-ɣ (pg/mL)	84 (81, 90)	82 (80, 90)	0.427
	TNF-α (pg/mL)	76 (68, 92)	76 (72, 90)	0.882
**ASDH**		**N = 55**	**ASDH, N = 15**	
	IL-10 (pg/mL)	252 (235, 272)	249 (236, 260)	0.909
	IL-1β (pg/mL)	28 (23, 32)	28 (27, 31)	0.909
	IL-6 (pg/mL)	145 (128, 177)	147 (128, 167)	0.909
	INF-ɣ (pg/mL)	83 (81, 90)	81 (76, 93)	0.909
	TNF-α (pg/mL)	79, 68, 93)	74 (62, 83)	0.909
**Cerebral oedema**		**N = 48**	**COE**, **N = 22**	
	IL-10 (pg/mL)	256 (235, 271)	240 (233, 260)	0.617
	IL-1β (pg/mL)	28 (24, 31)	29 (26, 33)	0.617
	IL-6 (pg/mL)	145 (130, 170)	148 (128, 178)	0.674
	INF-ɣ (pg/mL)	84 (81, 91)	81 (81, 88)	0.617
	TNF-α (pg/mL)	76 (63, 92)	81 (71, 93)	0.617
**Cerebral contusion**		**N = 61**	**CC, N = 9**	
	IL-10 (pg/mL)	249 (235, 270)	270 (236, 279)	0.366
	IL-1β (pg/mL)	28 (25 (25, 31)	29 (26, 33)	0.888
	IL-6 (pg/mL)	146 (142, 177)	127 (127, 157)	0.247
	INF-ɣ (pg/mL)	82 (81, 88)	92 (82, 93)	0.366
	TNF-α (pg/mL	79 (70, 93)	68 (62, 85)	0.366
**ICH**		**N = 60**	**ICH, N = 10**	
	IL-10 (pg/mL)	249 (234, 271)	263 (236, 270)	0.507
	IL-1β (pg/mL)	28 (25, 31)	32 (22, 47)	0.507
	IL-6 (pg/mL)	146 (130, 173)	142 (128, 168)	0.507
	INF-ɣ (pg/mL)	83 (81, 90)	81 (81, 86)	0.507
	TNF-α (pg/mL	79 (69, 93)	76 (62, 92)	0.507

EDH: Extradural Haemorrhage, ASDH: Acute Subdural Haemorrhage, CC: Cerebral Contusion, COE: Cerebral Oedema, ICH: Intracerebral Haemorrhage, TBI: Traumatic Brain Injury.

### Comparison of inflammatory cytokine concentrations and 6 months TBI outcome

**Fig. 1:**
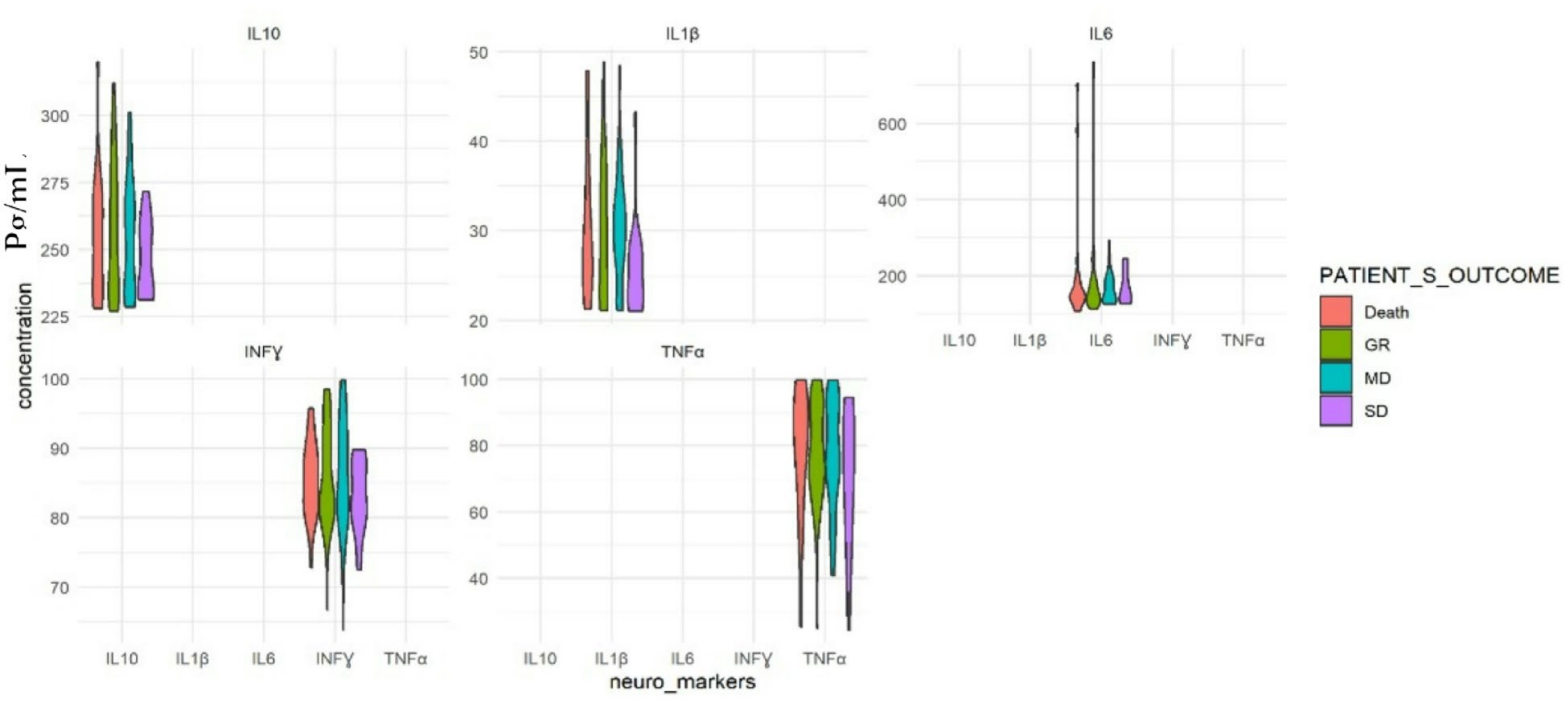
Violin plot showing Concentration of inflammatory cytokines according to 6 months outcome with GOSE. IL Interleukin, INF-ɣ: Interferon-gamma, TNF-α: Tumour necrosis factor-alpha, pg/mL: picogram/millilitre, Despite no significant association between concentrations of biomarkers and TBI outcome was found, this figure indicates, INF-ɣ, TNF-α had greater density values above 90 pg/mL, in TBI mortality compared to survival, where greater density values were found below 90pg/mL.

When the serum concentration of neuroinflammatory cytokines was associated with the 6-month outcome using the GOSE, no statistically significant association was found (*P* > 0.05) as seen in Fig. 1; Table 7.

**Table 7:** Concentration of inflammatory cytokines according to 6 months outcome withGOSE.

Characteristic	Death Median (25th percentile, 75th percentile)	GR Median (25th percentile, 75th percentile)	MD Median (25th percentile, 75th percentile)	SD Median (25th percentile, 75th percentile)	*P*-value
**N**	**22**	**59**	**55**	**16**	
IL-10 (pg/mL)	254 (236, 270)	256 (232, 279)	256 (235, 270)	245 (233, 257)	0.6
IL-1β (pg/mL)	28 (25, 35)	29 (23, 36)	30 (27, 34)	25 (22, 28)	0.056
IL-6 (pg/mL)	151 (142, 181)	149 (131, 187)	147 (131, 193)	149 (138, 203)	0.9
INF-ɣ (pg/mL)	86 (81, 89)	84 (81, 91)	85 (81, 92)	82 (80, 88)	0.4
TNF-α (pg/mL)	82 (70, 93)	75 (70, 92)	76 (71, 93)	73 (59, 86)	0.5

GR: Good recovery, MD: Moderate disability, SD: Severe Disability, IL Interleukin, INF-ɣ: Interferon-gamma, TNF—α: Tumour necrosis factor-alpha, pg/mL: picogram/millilitre.

### Comparison of inflammatory cytokine concentrations and 6 months TBI outcome (survival vs. mortality)

There was not a statistically significant difference in concentrations of cytokines, between mortality and survival (*p* > 0.05). However, patients who did not survive had higher values of IL-6 [151 (142, 184)]; INF-ɣ [86 (81, 89)], and TNF-α [82 (68, 93)], compared to survival: 149 (131, 193), 85 (81, 91), 76 (69, 92), respectively (Table 8).

**Table 8:** Comparison of inflammatory cytokine concentrations and 6 months TBI outcome (survival vs. mortality).

Characteristic	Mortality *N* = 22	Survival *N* = 130	*p*-value
IL-10 (pg/mL)	254 (235, 270)	254 (233, 271)	0.8
IL-1β (pg/mL)	28 (25, 36)	29 (23, 34)	0.9
IL-6 (pg/mL)	151 (142, 184)	149 (131, 193)	0.9
INF-ɣ (pg/mL)	86 (81, 89)	85 (81, 91)	0.9
TNF-α (pg/mL)	82 (68, 93)	76 (69, 92)	0.5

### Comparison of concurrent *T. gondii infection* and neuroinflammatory markers of TBI patients

**Fig. 2:**
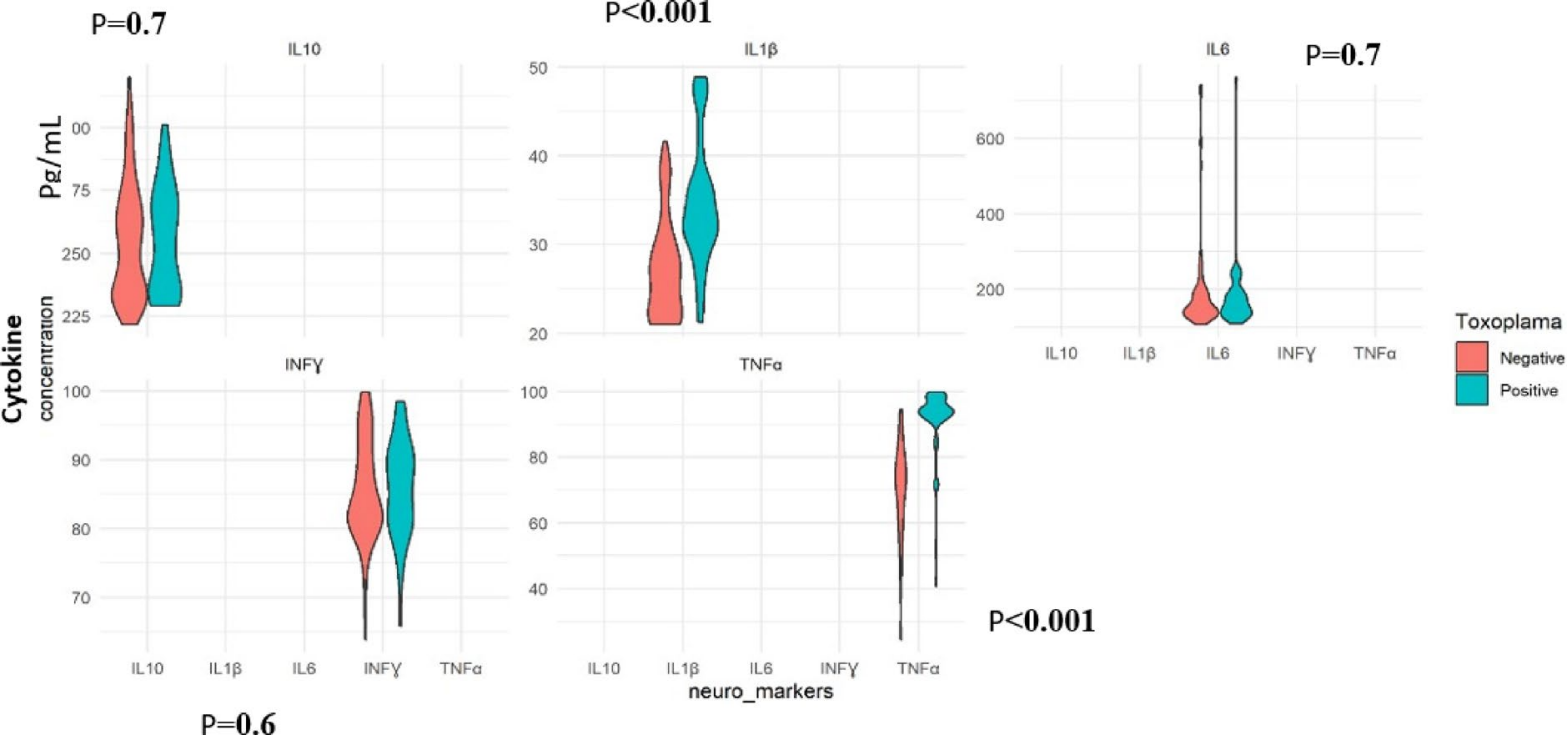
Concentration of inflammatory cytokines according to Toxoplasma positive and negative TBI cases. ***Significant at *P* < 0.001. IL Interleukin, INF-ɣ: Interferon-gamma, TNF-α: Tumour necrosis factor-alpha, pg/mL: picogram/millilitre. The figure indicates, IL-1β and TNF-α, have significantly greater density values, above [30 and 90]pg/mL, respectively, in TBI patients infected with *T. gondii*, compared to greater density values, below [30 and 90] pg/mL, respectively, for TBI patients seronegative to *T. gondii*.

An evaluation of the influence of *T. gondii* positivity on the concentrations of inflammatory cytokines revealed higher density values for all the *T. gondii* seropositive TBI cases, above 275 for IL-10, 30 for IL-1β, 200 for IL-6, 90 for TNF-α, and IFN-ɣ, compared to *T. gondii* seronegative cases, where high density values were below the above values, as shown in the violin plot below. However, this increase in neuro-markers concentrations in TBI patients seropositive to *T. gondii* was significant for IL-1β (*P* < 0.001) and TNF-α (*P* < 0.001), compared to TBI patients seronegative to *T. gondii* infection (Fig. 2).

### Age, sex, *T. gondii* infection and inflammatory marker’s concentrations between cases and healthy controls

Supplementary Table 2 shows that the mean age: 34(14), median age; 32(8,75) of cases did not vary significantly with the mean age; 30(8) and median age; 29(22, 56) of healthy controls (*p* = 0.3). Similarly, sex did not vary significantly among cases and controls (*p* = 0.2). *Toxoplasma gondii* infection was not detected in healthy controls [mean; 0.00(0.00) as compared to TBI cases [mean; 0.5(0.00, 1.93).

### Effect of *Toxoplasma gondii* positive neuroinflammatory markers on 6 months outcome with the GOSE (Favourable vs unfavourable outcome)

**Fig. 3:**
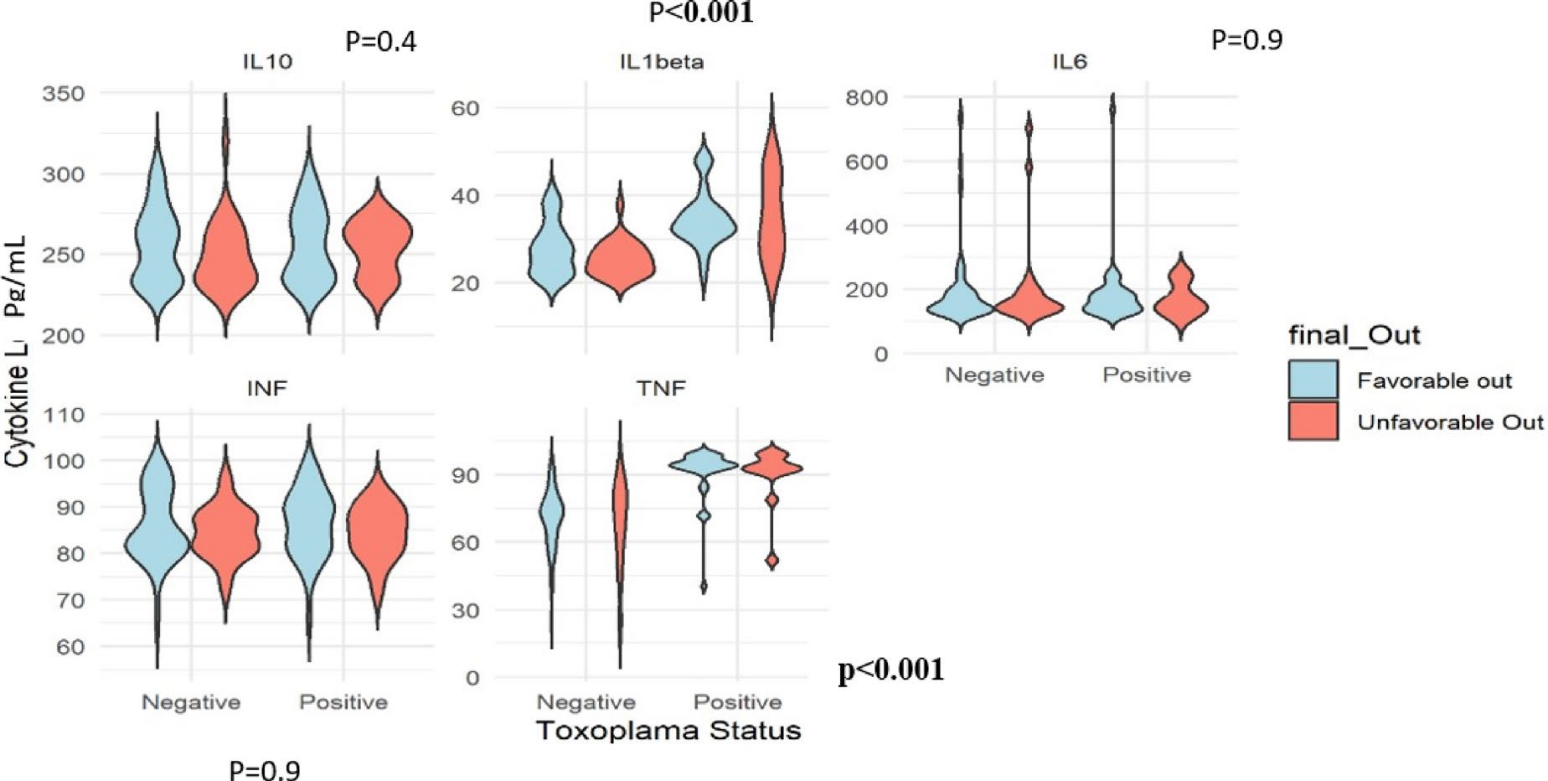
Comparison between *Toxoplasma gondii* positive versus negative neuroinflammatory marker concentrations with GOSE. ***Significant at p<0.001. IL: Interleukin, INF-ɣ: Interferon-gamma, TNF-α: Tumour necrosis factor-alpha, pg/mL: picogram/millilitre, Cytokine L: Cytokine Levels, Final Out: Final Outcome. IL-1β showing greater density values above 40pg/mL for unfavourable outcome in Toxoplasma positive TBI patients compared to Toxoplasma negative patients. Similarly, TNF-α shows higher density values above 90pg/mL for unfavourable outcome in Toxoplasma positive TBI patients compared to Toxoplasma negative patients, where higher density values are found below 90pg/mL.

We verified if TBI patients infected with latent *T. gondii* were more likely to have unfavourable outcomes 6 months after discharge from the hospital and presented in violin plots. TBI patients positive to *T. gondii* were more likely to have unfavourable outcomes reflected by significantly higher concentrations of IL-1β above 40 pg/mL (*p* < 0.001) and TNF-α above 90pg/mL (*p* < 0.001) compared to TBI patients seronegative to *T. gondii*, with greater densities below [40 and 90]pg/mL, respectively (Fig. 3).

### Comparison of *T. gondii* positive versus negative neuroinflammatory marker concentrations with GOSE in terms of survival vs mortality

The neuroinflammatory markers were further associated with *T. gondii* positive versus negative status and 6-month GOSE considering, survival versus non-survival. It was found that IL-10 had the highest concentration in *T. gondii* infected TBI patients who died [258 (236, 270)]. IL-1β concentration was significantly higher for *T. gondii*-infected TBI patients who did not survive [ 37 (31, 47) and 93 (88, 99) compared to [33 (31, 37) for TBI patients seropositive to *T. gondii*, who survived. TNF-α concentration was also high for *T. gondii* infected TBI patients who did not survive [93 (85, 99)], although also high for *T. gondii* infected patients who survived [94 (92, 96) as seen in Table 9.

**Table 9:** Comparison between *Toxoplasma gondii* positive versus negative neuroinflammatory marker concentrations with GOSE (mortality vs. survival).

Characteristic	Survival			Mortality		
	Negative Median (25th percentile, 75th percentile)	Positive Median (25th percentile, 75th percentile)	p-value	Negative Median (25th percentile, 75th percentile)	Positive Median (25th percentile, 75th percentile)	p-value
**N**	**82**	**40**		**26**	**12**	
IL-10 (pg/mL)	251 (233, 269)	256 (233, 272)	0.647	251 (232, 270)	258 (236, 270)	0.9203
IL-1β (pg/mL)	27 (22, 31)	33 (31, 37)	< 0.001	26 (23, 29)	37 (31, 47)	0.0145
IL-6 (pg/mL)	146 (130, 193)	161 (142, 194)	0.461	152 (142, 196)	150 (139, 198)	0.6027
INF-ɣ (pg/mL)	83 (81, 92)	87 (81, 91)	0.280	86.7 (81.8, 88.8)	85.4 (80.8, 89.8)	0.6027
TNF-α (pg/mL)	72 (62, 78)	94 (92, 96)	< 0.001	79 (62, 81)	93 (85, 99)	0.04729

IL: Interleukin, INF-ɣ: Interferon-gamma, TNF-α: Tumour necrosis factor-alpha, pg/mL: picogram/millilitre,

### Does *Toxoplasma gondii* infection independently predict mortality in TBI patients?

A multivariate logistic regression analysis was conducted to verify if *T. gondii* infection was associated with mortality. Although the odd ratio suggested a possible association between *T. gondii infection* and mortality in our cohort (OR = 2.010), this finding was not statistically significant (95% CI: 0.61–6.81, *p* = 0.3) as seen in Table 10.

**Table 10:** Association of *Toxoplasma gondii* status and mortality due to TBI.

Characteristic	OR	95% CI	*p*-value
**TBI severity**			
Mild	-	-	
Moderate	4.15	0.51, 86.0	0.2
Severe	69.3	12.2, 1338	< 0.001
***T. gondii*** **status**			
Negative	-	-	
Positive	2.01	0.61, 6.81	0.3
**Age**	1.02	0.98, 1.07	0.2

## Discussion

The aim of this prospective one-year cohort study at the Laquintinie Hospital of Douala was to present the inflammatory response in traumatic brain injury and to explore any potentiation of inflammation with concurrent infection with *T. gondii.* We also assessed the effects of inflammation on 6-month outcomes after TBI. Our findings reveal that all inflammatory markers analysed (IL-1β, IL-10, 1 L-6, TNF, IFN-γ) significantly increased in TBI patients compared with the healthy controls. This increase in inflammatory cytokines in TBI patients may reflect a neuroinflammatory status after traumatic brain injury or may this increase be attributed to a peripheral inflammatory process post TBI? However, it may reflect a neuroinflammatory process, as the TBI patients considered for the study had a healthy pre-injury state before sustaining brain trauma. On the other hand, the inflammation may be from other systemic factors.

About 33% of the TBI patients were seropositive to latent *T. gondii* infection. Of the 33% of TBI patients, infected with *Toxoplasma gondii*, the majority, 40 (77%) were within the age range of 15–45 years. This is contrary to reports which support the fact that *T. gondii* infection increases with age, and is very common in the elderly^42,43^. The high percentage of *T. gondii* infection in our study could be explained by several reasons; the sample (TBI patients) in majority were young adults, as this is the age at most risk of TBI, and only very few of the 160 TBI patients, belonged to the older age group. Also, the study was conducted in urban settings, where it is been reported to have higher transmission rates than in rural areas^44^. Douala is a highly cosmopolitan town in Cameroon, with a population of over 4.5 million inhabitants and is still faced with tremendous challenges in adequate drinking water provisions and in maintaining appropriate food safety for consumers. Furthermore, due to its massive population, disordered expansion and urbanization continue, increasing exposure to *T. gondii* infections. For these reasons, it is reported that living in an urban area is a risk factor for *Toxoplasma gondii* infection^44^. Moreover, there is an increasing trend of *T. gondii* infections in the younger population, which seems to level up to infections in the elderly population. This is demonstrated in the study of Morais et al.^44^ in Brazil, where young adults aged between 31 and 40 years represented 81% of *T. gondii* infection compared to those aged above 60 (90%).

TBI patients infected with *T. gondii*-induced had significantly higher values of IL-1β and TNF-α in the unfavourable and favourable outcomes 6 months after TBI. A previous study using the same cohort had highlighted significant higher levels of S100B in TBI patients seropositive to *Toxoplasma gondii*^45^.

The serum concentrations of the inflammatory cytokines evaluated in this study were increased in the TBI cases compared to the healthy controls, showing there was a possible inflammatory process ongoing after TBI. However, we cannot tell if this reflects a neuroinflammatory status, as cerebrospinal fluid was not assessed in this study. The reports by Kumar and Loane^10^and Rodney et al.^46^ showed increased levels of inflammatory cytokines. The increased serum levels of cytokines in TBI patients indicate alterations in systemic cytokine expression^47^. However, the concentrations of these cytokines did not significantly vary with TBI severity. This is not in line with the result from the study of Tsitsipanis et al.^48^, where levels of Interleukins 6 and 10 were significantly higher in severe TBI on day 1 for the adult group of TBI patients. This difference in results may be explained by the fact that our study did not consider cytokine concentrations per age group, contrary to the study of Tsitsipanis et al. where inflammatory cytokines were significantly high for severe TBI in the adult group and not in the paediatric group. Furthermore, the study of Aisiku et al.^49^ reported that IL-6, IL-8, and IL-10 values were significantly associated with acute respiratory distress syndrome in patients with severe TBI. This difference with our findings could be explained by the varying time points the biomarkers were tested post injury within the 24-hour post injury period, and that we assessed these biomarkers at just one time point. Furthermore, marked heterogeneity of pathoanatomical subtypes and diversity in the pathogenesis of traumatic brain injury contribute to differences in its course and outcomes^50^. Also, our small sample size (160 patients), could explain these differences in results. Although the concentration of cytokines was not significantly high in TBI patients with traumatic intracranial abnormality (*p* > 0.05), TNF-α levels were higher in patients with traumatic intracranial abnormality, compared to Normal CT. This is in line with the results obtained by Edwards et al.^51^ where concentrations of inflammatory cytokines were not significantly associated with traumatic intracranial abnormalities (CT-imaging) in mild TBI patients. Furthermore, the higher levels of TNF-α in patients with abnormal neuro-imaging results could be explained by its induction of apoptotic cell death (through p55 receptor activation) and exacerbation of secondary injury through neuroinflammation and excitotoxicity^52^. If cytokines are combined or grouped, could they provide better association with CT-imaging than when alone? Such results may aid especially in predicting the need for CT-imaging in mild TBI. Also, when inflammatory cytokines were associated with imaging location, all five cytokines showed varying levels with different types of traumatic brain injury. IL-10 had highest concentrations in patients with Cerebral contusion and intracerebral haemorrhage. IL-1β had highest concentrations in ICH, Cerebral contusion and cerebral oedema. IL-6 had highest concentration in extradural haemorrhage. INF-ɣ showed highest concentration in cerebral contusion, and TNF-α, highest in cerebral oedema. We can observe that INF-ɣ and TNF-α could likely be grouped to show generalised intracranial traumatic abnormality, while IL-1β and IL-10 showed mixed (general and focal) injuries, and IL-6 (focal traumatic intracranial injury). Further studies could examine combinations of inflammatory cytokines to better understand its effects on imaging location and using several neuro-imaging techniques.

When the serum concentration of inflammatory cytokines was correlated with the 6-month outcome using the GOSE, no significant association was found. However, it was noticed that IL-6, INF-ɣ, and TNF-α registered slightly higher values with mortality, like reports by Rodney et al.^46^ where IL-1β, IL-10, and IL-6 were found to be associated with the worst outcomes after TBI. These negative outcomes may partly be a consequence of the damage to neuronal activity which contributes to inhibiting neural regeneration and functional recovery. Therefore, early identification of neuroinflammation after TBI may trigger targeted interventions to reduce morbidity and mortality.

Furthermore, we demonstrate the effects of infection with obligate intracellular parasite; *Toxoplasma gondii* on inflammatory cytokines concentrations after traumatic brain injury. Concentrations of these cytokines were determined in TBI patients with concurrent *T. gondii* infection versus TBI patients seronegative to *T. gondii* and findings show the concentrations of IL-1β and TNF-α significantly increased in *T. gondii* positive TBI cases. Although it may be argued that this may not reflect specifically the manifestations of *T. gondii* infection of the central nervous system, as it may also result from a systemic effect of Toxoplasma co-infection, our findings are derived from TBI patients with normal pre-injury states. Reports by Estato et al.^17^ also stated that *T. gondii* infection reduces cerebral microvascular perfusion and induces neuroinflammation through activation of cerebral endothelial cells. This increase in serum concentrations of inflammatory cytokines is also supported by reports from Baker et al.^12^ who highlighted in a review the possible potentiation of neuroinflammation by concurrent *T. gondii* infection. Their review was however limited as no adequate conclusions could be drawn on the effects of *T. gondii* infection in traumatic brain injury in humans as no experiments were done. The significantly high levels of proinflammatory cytokines; IL-1β and TNF-α, in patients infected with *T. gondii*, could be explained by the fact that these markers are key actors in the proinflammatory response after TBI^53,54^. Furthermore, *T. gondii* induces the synthesis and release of IL-1β through activation of astrocytes and microglia^55^. IL-1β induces neuroinflammation in TBI through the inflammasome-induced caspase-1-mediated cleavage, in which IL-1β is synthesized in the CNS. This incremental and abnormal inflammatory stimulation leads to pyroptosis, potentially leading to even higher levels of IL-1β interstitially^56^. On the other hand, the significant increase in TNF-α may be due to its capacity to rapidly promote neuroinflammation in acute TBI, through direct or indirect potentiation of glutamate-mediated cytotoxicity mechanisms^57^. Furthermore, *T. gondii*-infected macrophages produce nitric oxide (NO) and tumor necrosis factor (TNF), which control replication of the intra-cellular parasite. A high TNF-α level contributes to neuroinflammatory response and can cause brain damage through triggering inflammatory cascade, disrupting blood-brain barrier and increasing excitotoxicity^58^. Also, the concentrations of the inflammatory cytokines might have been affected by different peak post-injury time rates, within 24 hours^54^. Therefore, considering peak time rates of the different markers and working with a larger population of TBI subjects, may provide more accurate results in future studies.” Findings from this study, therefore, constitutes the baseline for further research to better characterize *T. gondii*-induced pathology in TBI patients. This may help in the development or modification of diagnostic and therapeutic strategies in TBI care.

Considering the association of inflammatory cytokines concentrations and 6-month outcomes, findings from this study show that, although TBI cases had high serum concentrations of inflammatory cytokines, there were no effects on the 6-month outcome. Future research will focus on the effects of neuroinflammation on the outcome of TBI considering different time points in a longer follow-up. Neuroinflammation plays a non-negligible role in TBI pathology. Very little information exists on the association between inflammatory cytokines and outcomes of TBI. However, after TBI, there is an overlap of pathophysiology of these inflammatory cytokines in TBI, as it is reported that, the cellular cascade of inflammation could potentially have beneficial effects. However, if inflammation is too intense, prolonged, and unremitting, it may be harmful^59^. For example, pro-inflammatory cytokines aggravate and propagate neuroinflammation, degenerating healthy neurons and impairing brain functions^11^. This indicates that cytokine mediated inflammation plays an important role in secondary pathogenesis after TBI^60^. IL-1β is one of the most important causes of neuroinflammation post injury and has a role in glutamate excitotoxicity, neuronal degeneration and worsen TBI outcomes^61,53^. On the other hand, TNF-α has shown to possess both neurotoxic and neuroprotective activity. In TBI patients, TNF-α appears to be released faster than other proinflammatory cytokines and initiates the activation and recruitment of immune cells, induces cerebral inflammation, and dysregulation of the BBB breakdown^59^. Contrarily, the study by Loane et al.^62^ demonstrated that, lack of TNF-α, exacerbated tissue and BBB damage and impaired neurologic recovery after TBI in mice. To reconcile these conflicting reports of repair and damage, of these cytokines, Shohami et al.^63^, suggested that the exact timing and extent of TNF-α activation must be considered, as well as the presence of other cellular mediators. Similarly, IL-6 is also known for its dual-role in TBI, on one hand neurotoxic and causing secondary injury in TBI, as it promotes disruptions in the BBB and the progression of cerebral edema. On the other hand, it has neuroprotective effects through promoting neurogenesis and wound healing in animal models of TBI^64^. The complex interplay of several cellular factors, and consideration of post-injury specific peak time rates of the different cytokines may be considered in future studies, to draw accurate conclusions on the use of inflammatory markers to prognosticate TBI outcomes in Humans.

Furthermore, data are lacking on *T. gondii*-induced neuropathology in traumatic brain injury in humans. However, this has been studied in neurodegenerative diseases like Alzheimer’s disease by El-Saftawy et al.^65^ where they concluded that *T. gondii* immunopathological reactions can be a road paver to developing dementia. Furthermore, when inflammatory cytokines concentrations in the *T. gondii* infected and non-infected TBI cases were compared with the outcome, unfavourable outcomes were significantly associated with higher levels of IL-1β and TNF-α for TBI cases seropositive to *T. gondii.* Similarly, when associations with 6-month outcome were done in terms of mortality vs. survival, IL10, IL-1β, and TNF-α values were higher in *T. gondii* infected TBI patients who died. These are in line with reports by Baker et al.^12^ and Sun^66^who reported that concurrent *T. gondii* infection exacerbates neuroinflammatory response in TBI. This probably may increase the likelihood of *T. gondii*-infected TBI patients to have adverse TBI outcomes. Moreover, the levels of TNF-α, IL-6 and INF-ɣ, combined, were found to be significantly higher in positive CT scans after TBI as reported by Edwards et al.^51^, indicating these inflammatory cytokines are greatly involved in the pathomechanisms of TBI and its adverse outcome. Also, IL-1β has been implicated in secondary injury development after trauma and neutralizing its effects was reported to improve recovery following experimental traumatic brain injury^53^.

A multivariate logistic regression analysis was conducted to verify if *T. gondii* infection could predict mortality. Findings from this study show that TBI patients with latent infection of *Toxoplasma gondii* may be more likely to die, compared to TBI patients not infected with *T. gondii*, but this difference was not significant. Although studies that have reported associations between *T. gondii* infection and mortality after TBI are rare, Baker et al.^18^ reported exacerbation of abnormalities in *T. gondii* + TBI mice. Further multicentre studies with larger sample size on pre-existing *T. gondii* TBI patients may be important for TBI diagnosis, care and in development of better TBI prognostic models.

### Strength and limitations

The major strength of this study is that it is one of the rare studies that addressed inflammatory response in traumatic brain injury in humans. Furthermore, it adds up to the very few studies that have investigated *Toxoplasma gondii*-induced neuropathology in TBI patients, particularly in an African context where the spread of the feline parasite is on the rise^67^. The results of the study will add more insights into the understanding of inflammation in traumatic brain injury in positive or negative *T. gondii* patients. However, the study was limited by a few aspects; the sample size was small, thus did not permit us to generalise the findings. Also, the study did not assess inflammatory markers 6 months post-injury at the time we evaluated the outcome. Moreover, the peak post-injury time rates of the inflammatory cytokines, were not considered, and the 15 healthy control samples was small compared to the 160 patients. Another limitation of the study was that inflammatory markers were not assessed in Cerebrospinal Fluid (CSF). Therefore, other peripheric factors would have contributed to the increase in inflammatory cytokines assessed. Lastly, the healthy controls (*n* = 15) were a little age biased, as 14 were less than 46 years. Despite these limitations, the study is still scientifically relevant as it adds to knowledge on *T. gondii* co-infection in traumatic brain injury pathology, a field that has not been extensively exploited, especially in an African context.

### Conclusions and implications

*T. gondii* infection in TBI may have potentiated inflammatory profile through increase concentrations of inflammatory markers particularly IL-1β and TNF-α, in patients with concurrent *T. gondii* infection. *T. gondii*-infected TBI patients did not show significant adverse outcome compared to TBI patient not infected with *T. gondii.* These results indicate the need for further research on the enhancing role concurrent *T. gondii* infection has on neuroinflammation in traumatic brain injury and the potential use of pre-existing *T. gondii* infection for TBI care and prognostication. Further studies with larger sample sizes, long-term follow-up and inflammatory markers analysis at several time points, will provide more insights into *T. gondii*-induced neuroinflammation in TBI pathology and outcome. Also, future studies shall consider testing for neuroinflammation through biomarker analysis of the cerebrospinal fluid, which is the surest way to confirm inflammation in the Central Nervous System.

## Supplementary Information

Below is the link to the electronic supplementary material.

Supplementary Material 1

Supplementary Material 2

## Data Availability

The data sets used and/or analyzed during the current study are available from the corresponding author on reasonable request.

## Abbreviations

TBI: Traumatic Brain Injury
SSA: Sub-Saharan Africa
LMICs: Low-middle-income countries
IL: Interleukin
INF-ɣ: Interferon-gamma
TNF-α: Tumour necrosis factor-alpha
GOSE: Glasgow-coma outcome scale extended
CT: Computed tomography
LHD: Laquintinie hospital douala
GBD: Global burden of diseases

## References

[1] Wongchareon, K. et al. IMPACT and CRASH prognostic models for traumatic brain injury: external validation in a South-American cohort. *Inj Prev.* **26**(6), 546–554. 10.1136/injuryprev-2019-043466 (2020). 31959626 10.1136/injuryprev-2019-043466

[2] Dewan, M. C. et al. Estimating the global incidence of traumatic brain injury. *J. Neurosurg.* **130**, 1080–1097. 10.3171/2017.10.JNS17352 (2019). 29701556 10.3171/2017.10.JNS17352

[3] GBD 2016 Traumatic Brain Injury and Spinal Cord Injury Collaborators. Global, regional, and National burden of traumatic brain injury and spinal cord injury, 1990–2016: A systematic analysis for the global burden of disease study 2016. *Lancet Neurol.* **18**(1), P56–P87. 10.1016/S1474-4422(18)30415-0 (2018). 10.1016/S1474-4422(18)30415-0 PMC6291456 30497965

[4] Majdam, M. et al. Epidemiology of traumatic brain injuries in europe: A cross-sectional analysis. *Lancet Public. Health*. **1**(2), 76–83. 10.1016/S2468-2667(16)30017-2 (2016). 10.1016/S2468-2667(16)30017-2 29253420

[5] Buh, F. C. et al. Demographics, causes, and outcome of traumatic brain injury among trauma cases in Cameroon: A multi-center five year’s retrospective study. Neurotrauma Rep. **3**(1), 569–583 (2022). 10.1089/neur.2022.0053 PMC9879018 36711440

[6] Gardner, A. G. & Zafonte, R. Neuroepidemiology of traumatic brain injury. *Handb. Clin. Neurol.* **138**(3), 207–223. 10.1016/B978-0-12-802973-2.00012-4 (2016). 27637960 10.1016/B978-0-12-802973-2.00012-4

[7] Embolo, F. N. et al. Epidemiology of traumatic brain injury based on clinical symptoms among inhabitants of the South West region of cameroon: a community-based study. *Brain Inj*. **35**(10), 1184–1191 (2021). 34383629 10.1080/02699052.2021.1957150

[8] Adegboyega, G. et al. The burden of traumatic brain injury in Sub-Saharan africa: A scoping review. *World Neurosurg.* **156**, 192–205. 10.1016/j.wneu.2021.09.021 (2021). 10.1016/j.wneu.2021.09.021 34520864

[9] Dadas, A., Washington, J., Diaz-Arrastia, R. & Janigro, D. Biomarkers in traumatic brain injury (TBI): A review. *Neuropsychiatr Dis. Treat.* **8**, 14:2989–3000 (2018). 10.2147/NDT.S125620 PMC6231511 30510421

[10] Kumar, A. & Loane, D. J. Neuroinflammation after traumatic brain injury: Opportunities for therapeutic intervention. *Brain Behav. Immun.* **26**(8), 1191–1201 (2012). 22728326 10.1016/j.bbi.2012.06.008

[11] Hong, H., Kim, B. S. & Im, H. I. Pathophysiological role of neuroinflammation in neurodegenerative diseases and psychiatric disorders. *Int. Neurourol. J.* **20**(Suppl 1), S2–S7 (2016). 27230456 10.5213/inj.1632604.302 PMC4895907

[12] Baker, T. L. et al. Catastrophic consequences: can the feline parasite *Toxoplasma gondii* prompt the purrfect neuroinflammatory storm following traumatic brain injury? *J. Neuroinflammation*. **25**(17(1)), 222 (2020). 10.1186/s12974-020-01885-3 PMC7382044 32711529

[13] Corrigan, F., Mander, K. A., Leonard, A. V. & Vink, R. Neurogenic inflammation after traumatic brain injury and its potentiation of classical inflammation. J Neuroinflammation. 11;13(1):264 (2016). 10.1186/s12974-016-0738-9 PMC5057243 27724914

[14] Xiong, Y., Mahmood, A. & Chopp, M. Current Understanding of neuroinflammation after traumatic brain injury and cell-based therapeutic opportunities. *Chin. J. Traumatol.* **21**(3), 137–151 (2018). 29764704 10.1016/j.cjtee.2018.02.003 PMC6034172

[15] Mareze, M. et al. Socioeconomic vulnerability associated to *Toxoplasma gondii* exposure in Southern Brazil. *PLoS ONE*. **14**(2), e0212375 (2019). 30763391 10.1371/journal.pone.0212375 PMC6375698

[16] Baker, T. L. et al. Pre-existing *Toxoplasma gondii* infection increases susceptibility to pentylenetetrazol-induced seizures independent of traumatic brain injury in mice. *Front. Mol. Neurosci.* **15**, 1079097 (2023). 10.3389/fnmol.2022.1079097 PMC9849700 36683847

[17] Estato, V. et al. The neurotropic parasite *Toxoplasma gondii* induces sustained neuroinflammation with microvascular dysfunction in infected mice. *Am. J. Pathol.* **2188**(11), 2674–2687 (2018). 10.1016/j.ajpath.2018.07.007 30121257

[18] Baker, T. L. et al. A pre-existing *Toxoplasma gondii* infection exacerbates the pathophysiological response and extent of brain damage after traumatic brain injury in mice. *J. Neuroinflammation*. **21**, 14 (2024). 38195485 10.1186/s12974-024-03014-w PMC10775436

[19] Luo, P., Fei, F., Zhang, L., Qu, Y. & Fei, Z. The role of glutamate receptors in traumatic brain injury: Implications for postsynaptic density in pathophysiology. *Brain Res. Bull.* **85**(6), 313–320 (2011). 21605633 10.1016/j.brainresbull.2011.05.004

[20] Guerriero, R. M., Giza, C. C. & Rotenberg, A. Glutamate and GABA imbalance following traumatic brain injury. *Curr. Neurol. Neurosci. Rep.* **15**(5), 27 (2015). 25796572 10.1007/s11910-015-0545-1 PMC4640931

[21] Schimmel, S. J., Acosta, S. & Lozano, D. Neuroinflammation in traumatic brain injury: A chronic response to an acute injury. *Brain Circ.* **3**(3), 135–142 (2017). 30276315 10.4103/bc.bc_18_17 PMC6057689

[22] Gruenbaum, B. F., Zlotnik, A., Fleidervish, I., Frenkel, A. & Boyko, M. G. Neurotoxicity and destruction of the Blood-Brain barrier: Key pathways for the development of neuropsychiatric consequences of TBI and their potential treatment strategies. *Int. J. Mol. Sci.* **25**(17), 9628 (2022). 10.3390/ijms23179628 PMC9456007 36077024

[23] Carpio, A. et al. Parasitic diseases of the central nervous system: Lessons for clinicians and policy makers. *Expert Rev. Neurother.* **16**(4), 401–414 (2016). 26894629 10.1586/14737175.2016.1155454 PMC4926779

[24] Sribnick., E. A., Popovich, P. G. & Hall, M. W. Central nervous system injury-induced immune suppression. *Neurosurg. Focus*. **52**(2), E10 (2022). 35104790 10.3171/2021.11.FOCUS21586 PMC8931741

[25] Bouras, M., Asehnoune, K. & Roquilly, A. Immune modulation after traumatic brain injury. *Front. Med. (Lausanne)*. **9**, 995044 (2022). 36530909 10.3389/fmed.2022.995044 PMC9751027

[26] Griffin, G. D. The injured brain: TBI, mTBI, the immune system, and infection: Connecting the Dots. *Mil. Med.* **176**(4), 364–368 (2011). 21539156 10.7205/milmed-d-10-00021

[27] Sharma, R. et al. Infections after a traumatic brain injury: The complex interplay between the immune and neurological systems. *Brain. Behav. Immun.* **79**, 63–74 (2019). 31029794 10.1016/j.bbi.2019.04.034

[28] Daher, D. et al. Comprehensive overview of *Toxoplasma gondii*-Induced and associated diseases. *Pathogens* **20**(11), 1351 (2021). 10.3390/pathogens10111351 PMC8625914 34832507

[29] Jafari, M. M. et al. Immune system roles in pathogenesis, prognosis, control, and treatment of *Toxoplasma gondii* infection. *Int. Immunopharmacol.* **124**(Pt A), 110872 (2023). 10.1016/j.intimp.2023.110872 37660595

[30] Moghaddami, R., Mahdipour, M. & Ahmadpour, E. Inflammatory pathways of toxoplasmagondii infection in pregnancy. *Travel Med. Infect. Dis.* **62**, 102760 (2024). 39293589 10.1016/j.tmaid.2024.102760

[31] Konradt, C. et al. Endothelial cells are a replicative niche for entry of *Toxoplasma gondii* to the central nervous system. *Nat. Microbiol.* **15**(1), 16001 (2016). 10.1038/nmicrobiol.2016.1 PMC4966557 27572166

[32] Cabral, C. M. et al. Neurons are the primary target cell for the brain-tropic intracellular parasite *Toxoplasma gondii*. PLoS Pathog. **12**(2), e1005447 (2016). 10.1371/journal.ppat.1005447 PMC4760770 26895155

[33] Zheng, R. et al. Neuroinflammation following traumatic brain injury: take it seriously or not. *Front. Immunol.* **13**, 855701 (2022). 35392083 10.3389/fimmu.2022.855701 PMC8981520

[34] Eaton, J. et al. Epidemiology, management, and functional outcomes of traumatic brain injury in Sub-Saharan Africa. *World Neurosurg.* **108**, 650–655. 10.1016/j.wneu.2017.09.084 (2018). 10.1016/j.wneu.2017.09.084 28943422

[35] Benneh, G., Douala Cameroon & Britanica Consulted 30.03.2022. Available online: (2022). https://www.britannica.com/place/Cameroon (2022).

[36] World Population Review. Douala Population 2023. Available online (2023). https://worldpopulationreview.com/world-cities/doualapopulation

[37] Buh, F. C. et al. Traumatic brain injury in Cameroon: A prospective observational study in a level I trauma centre. *Medicina (Kaunas)*. **59**(9), 1558. (2023). 10.3390/medicina59091558 10.3390/medicina59091558 PMC10535664 37763678

[38] Czeiter, E. et al. Blood biomarkers on admission in acute traumatic brain injury: Relations to severity, CT findings and care path in the CENTER-TBI study. *EBioMedicine* **56**, 102785 (2020). 32464528 10.1016/j.ebiom.2020.102785 PMC7251365

[39] Ebrahimzadeh, A., Shahraki, M. K. & Mohammadi, A. Serological and molecular diagnosis of *Toxoplasma gondii* in patients with schizophrenia. *J. Parasit. Dis.* **42**(2), 177–181 (2018). 29844620 10.1007/s12639-018-0979-x PMC5962488

[40] Baird, A. C. et al. Dysregulation of innate immunity in ulcerative colitis patients who fail anti-tumor necrosis factor therapy. *World J. Gastroenterol.* **22**(41), 9104–9116 (2016). 27895398 10.3748/wjg.v22.i41.9104 PMC5107592

[41] Wu, D., Dinh, T. L., Bausk, B. P. & Walt, D. R. Long-term measurements of human inflammatory cytokines reveal complex baseline variations between individuals. *Am. J. Pathol.* **187**(12), 2620–2626 (2017). 28919109 10.1016/j.ajpath.2017.08.007

[42] Wilking, H., Thamm, M., Stark, K., Aebischer, T. & Seeber, F. Prevalence, incidence estimations, and risk factors of *Toxoplasma gondii* infection in germany: A representative, cross-sectional, serological study. *Sci. Rep.* **6**, 22551 (2016). 10.1038/srep22551 PMC4776094 26936108

[43] van den Berg, O. E. et al. Seroprevalence of *Toxoplasma gondii* and associated risk factors for infection in the netherlands: Third cross-sectional National study. *Epidemiol. Infect.* **151**, e136 (2023). 37503608 10.1017/S095026882300122X PMC10540174

[44] Morais, R. D. A. P. B., Carmo, E. L. D., Costa, W. S., Marinho, R. R. & Póvoa, M. M. *T. gondii* infection in urban and rural areas in the amazon: Where is the risk for toxoplasmosis? *Int. J. Environ. Res. Public. Health*. **17**(16), 8664 (2021). 10.3390/ijerph18168664 PMC8393968 34444413

[45] Buh, F. C. et al. Serum biomarker concentrations upon admission in acute traumatic brain injury: Associations with TBI Severity, *Toxoplasma gondii* Infection, and outcome in a referral hospital setting in Cameroon. *NeuroSci* **4**(3), 164–177 (2023). 39483201 10.3390/neurosci4030015 PMC11523680

[46] Rodney, T., Osier, N. & Gill, J. Pro- and anti-inflammatory biomarkers and traumatic brain injury outcomes: A review. *Cytokine* **110**, 248–256 (2018). 29396048 10.1016/j.cyto.2018.01.012

[47] LaPar, D. J. et al. Severe traumatic head injury affects systemic cytokine expression. *J. Am. Coll. Surg.* **214**(4), 478–486 (2012). discussion 486-8. 22342787 10.1016/j.jamcollsurg.2011.12.015 PMC3609411

[48] Tsitsipanis, C. et al. Inflammation biomarkers IL-6 and IL-10 May improve the diagnostic and prognostic accuracy of currently authorized traumatic brain injury tools. *Exp. Ther. Med.* **26**, 364 (2023). 37408863 10.3892/etm.2023.12063 PMC10318605

[49] Aisiku, I. P. et al. Plasma cytokines IL-6, IL-8, and IL-10 are associated with the development of acute respiratory distress syndrome in patients with severe traumatic brain injury. *Crit. Care*. **15**(20), 288 (2016). 10.1186/s13054-016-1470-7 PMC5024454 27630085

[50] Covington, N. V. & Duff, M. C. Heterogeneity is a hallmark of traumatic brain Injury, not a limitation: A new perspective on study design in rehabilitation research. *Am. J. Speech Lang. Pathol.* **30**(2S), 974–985 (2021). 33556261 10.1044/2020_AJSLP-20-00081

[51] Edwards, K. A. et al. Inflammatory cytokines associate with neuroimaging after acute mild traumatic brain injury. *Front. Neurol.* **11**, 348 (2020). 10.3389/fneur.2020.00348 PMC7248260 32508732

[52] Longhi, L. et al. Tumor necrosis factor in traumatic brain injury: Effects of genetic deletion of p55 or p75 receptor. *J. Cereb. Blood Flow. Metab.* **33**(8), 1182–1189 (2013). 23611870 10.1038/jcbfm.2013.65 PMC3734767

[53] Ozen, I. et al. Interleukin-1 beta neutralization attenuates traumatic brain injury-induced microglia activation and neuronal changes in the globus pallidus. *Int J Mol Sci.* **21**(2), 387 (2020). 10.3390/ijms21020387 PMC7014296 31936248

[54] Woodcock, T. & Morganti-Kossmann, M. C. The role of markers of inflammation in traumatic brain injury. *Front. Neurol.* **4**, 18 (2013). 23459929 10.3389/fneur.2013.00018 PMC3586682

[55] French, T. et al. Neuronal impairment following chronic *Toxoplasma gondii* infection is aggravated by intestinal nematode challenge in an IFN-γ-dependent manner. *J. Neuroinflammation*. **16**(1), 159. 10.1186/s12974-019-1539-8 (2019). 31352901 10.1186/s12974-019-1539-8 PMC6661741

[56] Lindblad, C., Rostami, E. & Helmy, A. Interleukin-1 receptor antagonist as therapy for traumatic brain injury. *Neurotherapeutics* **20**(6), 1508–1528 (2023). 37610701 10.1007/s13311-023-01421-0 PMC10684479

[57] Olmos, G. & Lladó, J. Tumor necrosis factor alpha: A link between neuroinflammation and excitotoxicity. *Mediators Inflamm*. **2014**, 861231 (2014). 10.1155/2014/861231 PMC4055424 24966471

[58] French, T. et al. Neuronal impairment following chronic *Toxoplasma gondii* infection is aggravated by intestinal nematode challenge in an IFN-γ-dependent manner. *J. Neuroinflammation*. **16**(1), 159. 10.1186/s12974-019-1539-8 (2019). 31352901 10.1186/s12974-019-1539-8 PMC6661741

[59] Postolache, T. T. et al. Inflammation in traumatic brain injury. *J. Alzheimers Dis.* **74**(1), 1–28 (2020). 32176646 10.3233/JAD-191150 PMC8190673

[60] Thelin, E. P. et al. Elucidating pro-inflammatory cytokine responses after traumatic brain injury in a human stem cell model. *J. Neurotrauma*. **35**(2), 341–352 (2018). 28978285 10.1089/neu.2017.5155 PMC5784793

[61] Ziebell, J. M., & Morganti-Kossmann, M. C. Involvement of pro-and anti-inflammatory cytokines and chemokines in the pathophysiology of traumatic brain injury. *Neurotherapeutics* **7**(1), 22–30 (2010). 20129494 10.1016/j.nurt.2009.10.016 PMC5084109

[62] Loane, D. J., Stoica, B. A. & Faden A.I. Neuroprotection for traumatic brain injury. *Handb. Clin. Neurol.* **127**, 343–366 (2015). 25702227 10.1016/B978-0-444-52892-6.00022-2 PMC4808298

[63] Shohami, E., Ginis, I. & Hallenbeck, J. M. Dual role of tumor necrosis factor alpha in brain injury. *Cytokine Growth Factor Rev.* **10**(2), 119–130 (1999). 10743503 10.1016/s1359-6101(99)00008-8

[64] Ciryam, P., Gerzanich, V. & Simard, J. M. Interleukin-6 in traumatic brain injury: A Janus-faced player in damage and repair. *J. Neurotrauma*. **40**(21–22), 2249–2269 (2023). 37166354 10.1089/neu.2023.0135 PMC10649197

[65] El Saftawy, E. A. et al. Can *Toxoplasma gondii* pave the road for dementia? *J. Parasitol. Res.* **30**, 8859857 (2020). 10.1155/2020/8859857 PMC7414348 32802484

[66] Sun, M. The Effect of *Toxoplasma gondii* Infection on Common Acquired Brain Insults. Project, Monash University 2022. Available online (2022). https://brainfoundation.org.au/research-grants/2019/neural-infections/

[67] Bokaba, R. P. et al. *Toxoplasma gondii* in African wildlife: A systematic review. *Pathogens* **11**(8), 868 (2022). 36014989 10.3390/pathogens11080868 PMC9414955

