# Supplementary material for: Concurrent Toxoplasma gondii infection and neuroinflammation in traumatic brain injury patients in a referral hospital in Douala Cameroon
Source: Sci Rep. 2026 Mar 12;16:13308. doi: 10.1038/s41598-026-40284-1 (PMC13106797; doi:10.1038/s41598-026-40284-1)
Supplement: Supplementary file 1 — Supplementary Material 1 [file 41598_2026_40284_MOESM1_ESM.pdf]

Supplementary material

Supplementary Table 1: Concentration of inflammatory markers according to post injury time arrival.

**Characteristic**	**<12 h**<br>**Median (IQR)**	**12-24 h**<br>**Median (IQR)**	**P-value**
**N**	**141**	**19**	
IL10 (pg/mL)	255 (235, 271)	252 (232, 268)	0.6
IL1β (pg/mL)	29 (24, 34)	28 (26, 36)	0.5
IL6 (pg/mL)	149 (131, 193)	156 (142, 191)	0.8
INFɣ (pg/mL)	85 (81, 91)	83 (81, 91)	0.8
TNFα (pg/mL)	76 (69, 92)	79 (69, 92)	0.9
