# Supplementary material for: Concurrent Toxoplasma gondii infection and neuroinflammation in traumatic brain injury patients in a referral hospital in Douala Cameroon
Source: Sci Rep. 2026 Mar 12;16:13308. doi: 10.1038/s41598-026-40284-1 (PMC13106797; doi:10.1038/s41598-026-40284-1)
Supplement: Supplementary file 2 — Supplementary Material 2 [file 41598_2026_40284_MOESM2_ESM.pdf]

Supplementary Table 2: Age, sex, *T. gondii* infection and inflammatory marker concentrations between cases and healthy controls

**Characteristic**	**Overall N=175**	**Cases; n=160**	**Healthy controls N=15**	**p-value**
**Age**				0.3
Mean (SD)	34(13)	34(14)	30(8)	
Median (Min, Max)	31 (8, 75)	32(8,75)	29(22, 56)	
**Sex**				0.2
Male	19 (11%)	16 (10%)	3(20%)	
Female	156 (89%)	144 (90%)	12(80%)	
T. gondii				0.006
Mean (SD)	0.45(0.7)	0.5(0.72)	0.0(0.0)	
Median (Min, Max)	0.0(0.0, 1.93)	0.0(0.0, 1.93)	0.0(0.0)	
**IL-10 (pg/mL)**				<0.001
Mean (SD)	236(65)	255(22)	37(5)	
Median (Min, Max)	249(32, 320)	254(222, 320)	37(32, 45)	
**IL-1β (pg/mL)**				<0.001
Mean (SD)	28(9)	30(7)	7(1)	
Median (Min, Max)	28(6, 49)	29(21, 49)	7(6, 10)	
**IL-6 (pg/mL)**				<0.001
Mean (SD)	173(120)	189(114)	10(1)	
Median (Min, Max)	147(9, 762)	149(107, 762)	10(9, 12)	
**INF-ɣ (pg/mL)**				<0.001
Mean (SD)	81(18)	86(7)	28(5)	
Median (Min, Max)	83(22, 100)	85(64, 100)	28(22, 43)	
**TNF-α (pg/mL)**				<0.001
Mean (SD)	71(26)	77(17)	6(1)	
Median (Min, Max)	75(5, 100)	77(24, 100)	6(5, 7)	
